# Supplementary material for: Comparative Efficacy of Tocilizumab and Baricitinib Administration in COVID-19 Treatment: A Retrospective Cohort Study
Source: Medicina (Kaunas). 2022 Apr 4;58(4):513. doi: 10.3390/medicina58040513 (PMC9030469; doi:10.3390/medicina58040513)
Supplement: Supplementary file 1 [file medicina-58-00513-s001.zip › Table S1.pdf]

Table S1. Predicting risk factors for infection event within 28 days of treatment with tocilizumab or baricitinib.

	univariate	
	OR (95%CI)	*P*-value
Characteristics		
Baricitinib use	0.93 (0.29-2.98)	0.904
Age (year)	1.03 (0.98-1.08)	0.208
Sex(male)	0.63 (0.19-2.08)	0.453
Current smoker	0.75 (0.15-3.74)	0.728
BMI ≧ 30 (kg/m^2)	0.69 (0.18-2.73)	0.694
Chronic heart disease	2.96 (0.86-10.18)	0.085
Diabetes mellitus	0.83 (0.26-2.68)	0.767
Hypertension	2.61 (0.82-8.31)	0.104
Time from onset to administration ≦ 7day	2.43 (0.79-7.48)	0.123
Treatment		
Heparin	0.66 (0.18-2.33)	0.657
Any anti-viral drug	0.55 (0.17-1.83)	0.332
Blood test at administration		
Lymphocytes (×10^3/μL)	0.97 (0.82-1.15)	0.747
Platelet (×10^5/μL)	0.88 (0.50-1.56)	0.672
LDH (×10^{2}U/L)	1.10(0.82-1.49)	0.498
CRP (mg/mL)	1.00 (0.90-1.10)	0.897
KL-6 (×10^{2}U/mL)	1.08 (0.91-1.29)	0.373
Procalcitonin (ng/mL)	1.22 (0.17-8.46)	0.844
Ferritin (ng/mL)	1.11 (0.65-1.88)	0.706
D-dimer (μg/mL)	1.02 (1.00-1.04)	0.060

BMI, body mass index; CI, Confidence interval; CRP, C-reactive protein; KL-6, Krebs von den Lungen-6; LDH, lactate dehydrogenase; OR, odds ratio.
