# Supplementary material for: Comparative Efficacy of Tocilizumab and Baricitinib Administration in COVID-19 Treatment: A Retrospective Cohort Study
Source: Medicina (Kaunas). 2022 Apr 4;58(4):513. doi: 10.3390/medicina58040513 (PMC9030469; doi:10.3390/medicina58040513)
Supplement: Supplementary file 1 [file medicina-58-00513-s001.zip › Table S2.pdf]

Table S2. Comparison between baseline characteristics of tocilizumab and baricitinib groups (after propensity score matcing).

	Tocilizumab (n=25)	Baricitinib (n=25)	*P*-value
Characteristics			
Age (year)	60 (54, 66)	59 (53, 66)	0.75
Sex (male)	17 (68.0)	20 (80.0)	0.33
Current smoker	7 (28.0)	4 (16.0)	0.31
BMI ≧ 30 (kg/m^2)	9 (36.0)	6 (25.0)	0.40
Chronic heart disease	5 (20.0)	1 (4.0)	0.082
Chronic kidney disease	0 (0)	2 (8.0)	0.15
Diabetes mellitus	10 (40.0)	10 (40.0)	1.00
Any collagen disease	1 (4.0)	0 (0)	0.31
Hypertension	12 (48.0)	9 (42.9)	0.39
Any respiratory disease	1 (4.0)	2 (8.0)	0.55
Immunosuppressive drug regular use	0 (0)	1 (2.0)	0.31
Vaccination twice	0 (0)	1 (2.0)	0.31
Time from symptom onset to administration	9 (8, 13)	9 (7, 11)	0.39
Time from onset to administration ≦ 7day	8 (32.0)	6 (24.0)	0.53
Treatment			
Steroid	25 (100)	25 (100)	N/A
Heparin	21(84.0)	15 (60.0)	0.059
Any anti-viral drug	22 (88.0)	21 (84.0)	0.68
Antibody combination casirivimab/imdevimab	0 (0)	1 (1.0)	0.144
Severity			
1	0 (0)	0 (0)	
2	2 (8.0)	5 (20.0)	
3	18 (72.0)	19 (76.0)	
4	5 (20.0)	1 (4.0)	0.137
Outcomes			
Death within 28 days	2 (8.0)	1 (4.0)	0.55
Improvement in respiratory status within 28 days	21 (84.0)	22 (88.0)	0.68
Development of secondary infections	3 (12.0)	4 (16.0)	0.68

Data are shown as median (interquartile range) or number (%).

We categorized COVID-19 severity at treatment initiation as follows; severity level 1: hospitalized but not requiring supplemental oxygen; severity level 2: hospitalized and requiring supplemental oxygen ≤ 4 L/min; severity level 3: hospitalized and requiring oxygen therapy ≥ 5 L/min or re-ceiving nasal high-flow oxygen therapy, non-rebreather, or noninvasive mechanical ventilation; and severity level 4, receiving invasive mechanical ventilation.

BMI, body mass index; CRP, C-reactive protein; KL-6, Krebs von den Lungen-6; LDH, lactate dehydrogenase.
